# Mindfulness-based interventions for perinatal depression, anxiety, and stress: a systematic review and meta-analysis of randomized controlled trials, with an exploratory subgroup signal by delivery mode

**DOI:** 10.3389/fpsyt.2026.1868153

**Published:** 2026-08-04

**Authors:** Gaoqin Tang, Unfai Cho, Jun Zhang, Min Chen

**Affiliations:** 1 Faculty of Chinese Medicine and State Key Laboratory of Mechanism and Quality of Chinese Medicine, Macau University of Science and Technology, Macao, Macao SAR, China; 2 Macau University of Science and Technology (M.U.S.T.) Innovation Technology Research Institute, Zhuhai, China

**Keywords:** digital health (mHealth), meta-analysis, mindfulness-based interventions, perinatal depression, randomized controlled trials

## Abstract

**Background:**

Perinatal depression is a highly common clinical complication. While expert panels advocate for non-pharmacological treatments, the relative benefits of mindfulness-based interventions delivered face-to-face versus through emerging digital platforms have not been examined within a single integrated synthesis that distinguishes the contribution of delivery mode from that of trial era and methodology.

**Methods:**

We conducted a systematic review and meta-analysis of parallel-group randomized controlled trials evaluating mindfulness-based interventions for perinatal women. The synthesis relied exclusively on directly verifiable, publicly accessible study-level data, obtained from primary full-text reports and, for seven of the ten depression trials, from the open controlled-data table of a prior peer-reviewed meta-analysis (Lever Taylor et al., 2016). The primary outcome was post-intervention depressive symptoms, with anxiety and stress as secondary outcomes. A pre-specified subgroup analysis examined delivery mode (digital versus face-to-face) as an exploratory, hypothesis-generating moderator rather than as a head-to-head comparison.

**Results:**

Ten RCTs (591 perinatal women) contributed depression data. MBIs significantly reduced depressive symptoms (Hedges g = -0.31, 95% CI -0.48 to -0.14; P < .001; I² = 0%), with a 95% prediction interval excluding the null. In an exploratory, pre-specified subgroup analysis, the digital subgroup showed a larger pooled effect than the face-to-face subgroup (g = -0.40 vs. -0.13; test for subgroup differences, P = .04). Because delivery mode was completely confounded with publication year, trial size, and risk of bias, and because all three digital trials enrolled antenatal samples, this difference is reported as hypothesis-generating rather than as evidence of modality superiority. Pooled effects favored MBIs for anxiety (g = -0.29; k = 8) but were not significant for stress (g = -0.11; k = 8). Sensitivity analyses supported the robustness of the depression finding.

**Conclusion:**

MBIs produce a small-to-moderate reduction in perinatal depressive and anxiety symptoms, with a numerically larger depression effect in the digital subgroup. Because delivery mode is completely confounded with publication year, trial size, and risk of bias in the present evidence base, this subgroup difference is hypothesis-generating, and no delivery mode can yet be considered superior. Confirmatory trials using active comparators, longer postpartum follow-up, and standardized outcome reporting are needed before any delivery mode can be considered superior.

## 1 Introduction

Perinatal depression, defined as depressive symptoms occurring during pregnancy or within the first 12 months postpartum, is among the most common clinical complications of this period. Pooled prevalence has been estimated at approximately 24.7% in low- and middle-income countries (1), with broadly comparable, though generally somewhat lower, figures reported across high-income settings (2). Reported estimates vary substantially by region, sampling frame, and assessment instrument. The burden is unevenly distributed; the highest rates are observed in vulnerable rural populations, where prevalence has been reported to exceed 25% (3). The downstream consequences extend beyond the affected mother: untreated perinatal depression has been linked to disrupted mother–infant bonding (4), and, more broadly, to impairments in child cognitive and socioemotional development, increased child behavioral difficulties, and shorter breastfeeding duration (5).

Selective serotonin reuptake inhibitors remain a cornerstone of pharmacological treatment for perinatal depression; however, concerns regarding fetal and breast-milk drug exposure, patient preference for non-pharmacological options, and limited access to specialist mental-health services collectively contribute to a substantial treatment gap (6). Expert panels have therefore advocated for the broader integration of scalable, accessible, non-pharmacological alternatives within routine perinatal care (6). In this context, mindfulness-based interventions (MBIs) have attracted considerable attention. These interventions include Mindfulness-Based Stress Reduction (MBSR), Mindfulness-Based Cognitive Therapy (MBCT), Mindfulness-Based Childbirth and Parenting (MBCP), and newer hybrid programs that combine mindfulness training with cognitive-behavioral or psychoeducational components.

The proposed mechanisms underlying the benefits of MBIs in perinatal mental health include attenuation of hypothalamic-pituitary-adrenal (HPA) axis reactivity, reductions in rumination and experiential avoidance, enhanced interoceptive awareness, and improved emotion regulation (7, 8). Biological plausibility is reinforced by a randomized controlled trial in pregnant Chinese women in which a mindfulness-based psychosomatic intervention significantly reduced both pregnancy-related stress and HPA-axis cortisol output (8). From an empirical standpoint, previous meta-analyses have reported small-to-moderate effects of MBIs on perinatal depression, anxiety, and stress, with the magnitude of pooled effects varying as a function of eligibility criteria, outcome instruments, and the choice of comparator (9, 10). A 2023 meta-analysis of 25 RCTs (n = 2,495) reported that MBIs were associated with greater reductions in perinatal depression and anxiety than control conditions, with benefits sustained into the postpartum period (9).

Over the past five years, the proliferation of digital mindfulness modalities, such as smartphone applications, self-paced online courses, and web-based curricula, has emerged as a strategy to overcome long-recognized barriers to in-person delivery, including time constraints, stigma, cost, geographical access, and feasibility for postpartum women (11). A 2023 meta-analysis of digital mindfulness interventions in pregnancy reported statistically significant reductions in depressive (Hedges *g* = –0.47) and anxiety (Hedges *g* = –0.41) symptoms, although stress outcomes were less consistent (12). Subsequent systematic reviews have continued to support the feasibility and acceptability of digital perinatal MBIs while underscoring the need for higher-quality, more reproducible evidence (13–16).

Despite this expanding literature, three substantive gaps remain. First, few meta-analyses have examined face-to-face and digital delivery within a single integrated synthesis, although, as we emphasize below, any such contrast within the current evidence base is necessarily exploratory, because delivery mode is confounded with trial era and methodology. Second, the existing evidence base is dominated by older, smaller pilot RCTs, whereas newer and comparatively larger digital RCTs have not yet been comprehensively incorporated. Third, reproducibility concerns have arisen with respect to meta-analyses whose study-level inputs cannot be verified against the original reports or against publicly accessible data sources. The present review addresses these gaps by synthesizing parallel-group RCTs of MBIs in perinatal women using only study-level data that are directly verifiable against publicly accessible sources, namely the primary full-text reports or an open controlled-data table of a prior peer-reviewed meta-analysis. Depressive symptoms were specified as the primary outcome, and anxiety and stress as secondary outcomes. A pre-specified subgroup analysis contrasting face-to-face and digital delivery was incorporated to explore, in a hypothesis-generating manner, whether the magnitude of MBI effects appears to differ by mode of delivery.

## 2 Methods

### 2.1 Protocol and reporting

This systematic review and meta-analysis was designed and reported in accordance with the Preferred Reporting Items for Systematic Reviews and Meta-Analyses (PRISMA) 2020 statement (17). A review protocol specifying the research question, eligibility criteria, target databases, search and screening strategy, data-extraction procedures, risk-of-bias assessment, and pre-specified statistical analyses was prepared in advance of the literature search. Any deviations from the protocol are documented in the relevant subsection. Because the synthesis was based exclusively on previously published, aggregate-level data, ethical approval and informed consent were not required.

The review question was framed using the PICOS structure: Population, perinatal women (pregnant or up to 12 months postpartum); Intervention, manualized mindfulness-based interventions delivered in person or via digital platforms; Comparator, usual care, waitlist, attention control, or other non-mindfulness control conditions; Outcomes, post-intervention depressive (primary), anxiety, and stress symptoms quantified with validated instruments; Study design, parallel-group randomized controlled trials.

### 2.2 Eligibility criteria

Eligibility was defined *a priori* according to the PICOS framework, with each criterion applied uniformly at the title-and-abstract and full-text screening stages. To facilitate independent application, the criteria were operationalized as discrete inclusion and exclusion items, presented below.

Inclusion criteria

- Study design: parallel-group randomized controlled trials.
- Population: women aged 18 years or older in the perinatal period, defined as pregnancy through 12 months postpartum. Mixed-sex or mixed-population studies were eligible only when data for the perinatal female subgroup could be disaggregated.
- Intervention: structured, manualized mindfulness-based programs, including but not limited to MBSR, MBCT, MBCP, and hybrid curricula, delivered in person (individual or group) or digitally (smartphone application, self-directed web module, or internet-supported program). Mindfulness was required to be the principal therapeutic component.
- Comparator: treatment as usual, routine antenatal or postnatal care, waitlist, attention control, active non-mindfulness control (e.g., childbirth education, self-monitoring), or no intervention.
- Outcomes: post-intervention assessment of at least one of three continuous outcomes using a validated instrument, namely depression (EPDS, CES-D, CESD-R, DASS-21 depression subscale, or Beck Depression Inventory), anxiety (GAD-7, State-Trait Anxiety Inventory, or DASS-21 anxiety subscale), or stress (Perceived Stress Scale or DASS-21 stress subscale).
- Data availability: arm-level post-intervention means and standard deviations directly retrievable online, either from the primary full-text publication or from an open controlled-data table of a prior meta-analysis.
- Language and time frame: peer-reviewed articles published in English or Chinese between 1 January 2008 and 28 February 2026.

Exclusion criteria

- Cluster-randomized trials, crossover trials, quasi-experimental studies, uncontrolled before-after studies, case reports, narrative reviews, conference abstracts without an accompanying full text, and stand-alone protocol papers.
- Mixed-population studies in which female perinatal participants could not be disaggregated.
- Trials in which mindfulness was not the principal therapeutic component (e.g., predominantly cognitive-behavioral or psychoeducational programs with mindfulness as an ancillary element).
- Studies for which arm-level numeric outcome data were not directly accessible in a manner consistent with open-science verification principles.

The lower temporal bound of January 2008 was selected deliberately to coincide with the publication year of the earliest perinatal MBI RCT meeting our criteria; restricting to records before this date would not have yielded additional eligible trials.

### 2.3 Information sources and search strategy

Eight electronic databases were searched for records indexed between 1 January 2008 and 28 February 2026: PubMed, Embase, the Cochrane Central Register of Controlled Trials (CENTRAL), Web of Science Core Collection, PsycINFO, CINAHL, the China National Knowledge Infrastructure (CNKI), and Wanfang Data. To complement the database searches, the reference lists of relevant systematic reviews and of every included primary study were hand-searched, and ClinicalTrials.gov was screened for completed trials not yet indexed in the principal databases.

The strategy was constructed around three concept blocks, namely population, intervention, and outcome, joined with the Boolean operator AND. The intervention block combined a set of core mindfulness terms with a dedicated digital and mHealth supplement, and both the core and the digital terms were applied uniformly across all eight databases. Where the database offered a validated randomized controlled trial filter, this was applied to restrict retrieval to randomized designs. The complete sets of search terms used in each block are listed below.

Population terms

- perinatal, pregnancy, pregnant, antenatal, prenatal, postpartum, postnatal, maternal.

Intervention terms (core)

- mindfulness, MBSR, MBCT, MBCP, meditation, mindful.

Intervention terms (digital and mHealth supplement)

- app, mobile, digital, mHealth, web-based, online, eHealth, internet, smartphone.

To illustrate operationalization, the executed PubMed string was structured as: (perinatal[tiab] OR pregnancy[tiab] OR pregnant[tiab] OR antenatal[tiab] OR prenatal[tiab] OR postpartum[tiab] OR postnatal[tiab] OR maternal[tiab]) AND (mindfulness[tiab] OR MBSR[tiab] OR MBCT[tiab] OR MBCP[tiab] OR meditation[tiab] OR mindful[tiab] OR app[tiab] OR mobile[tiab] OR digital[tiab] OR mHealth[tiab] OR web-based[tiab] OR online[tiab] OR eHealth[tiab] OR internet[tiab] OR smartphone[tiab]) AND (depression[tiab] OR anxiety[tiab] OR stress[tiab]) AND (randomized[tiab] OR randomised[tiab] OR RCT[tiab]). The digital and mHealth terms shown here within the intervention block were part of the executed search in every database; an earlier displayed version of this illustrative string had inadvertently omitted them. This strategy retrieved 687 records from PubMed, with database-specific syntactic adaptations of the same logic yielding 821 records from Embase, 412 from CENTRAL, 736 from Web of Science, 398 from PsycINFO, 265 from CINAHL, 187 from CNKI, and 148 from Wanfang. The complete database-specific search strategies, the extracted arm-level data, and the annotated R analysis scripts are provided as **Supplementary Files** accompanying this article.

The search strategy was developed with reference to established perinatal mental-health systematic reviews and was internally peer-reviewed prior to execution to ensure comprehensive coverage of the eligible literature.

### 2.4 Study selection

Records retrieved from all sources were imported into EndNote 21 (Clarivate, Philadelphia, PA, USA) for deduplication and were subsequently transferred to Covidence (Veritas Health Innovation, Melbourne, Australia) for managed screening. Two reviewers independently applied the eligibility criteria to titles and abstracts; records judged potentially eligible by either reviewer advanced to full-text assessment. The same two-reviewer procedure was applied at the full-text stage, with reasons for exclusion documented for every excluded study. Disagreements were resolved through discussion and, when necessary, adjudicated by a senior reviewer. Inter-rater agreement at the full-text stage was substantial (Cohen’s κ = 0.87).

### 2.5 Data extraction

A purpose-built extraction form was developed in Microsoft Excel, piloted on two trials, and refined before being applied to the full pool of eligible studies. Extraction was performed in parallel: one reviewer entered the data and a second reviewer independently verified each entry. Extracted variables were organized into six domains: bibliographic information (author, year, country); population characteristics (gestational age, postpartum window, parity, risk profile, and sample size per arm); intervention specifications (type of MBI, duration, mode of delivery, number and length of sessions, and facilitator training); comparator condition; timing of outcome assessment; and outcome instruments together with arm-level means and standard deviations contributing to the depression, anxiety, and stress syntheses.

Where post-intervention means and standard deviations were not directly reported but could be derived from other summary statistics (e.g., medians and interquartile ranges, or means with 95% confidence intervals), standard transformations were applied prior to inclusion. When a single trial reported multiple eligible instruments for the same outcome, a pre-specified instrument hierarchy was used to minimize cross-trial measurement heterogeneity (depression: EPDS > CESD-R > CES-D > DASS depression subscale, in either its original 42-item or its 21-item form > Beck Depression Inventory; anxiety: GAD-7 > STAI > DASS-21 anxiety subscale; stress: PSS > DASS-21 stress subscale). The hierarchy was made fully consistent with the list of eligible instruments specified in the inclusion criteria; the original 42-item DASS depression subscale (used by Perez-Blasco et al., 2013) and the Beck Depression Inventory were retained as *a priori* eligible instruments, although in the event no included trial reported a depression outcome on the Beck Depression Inventory.

Authors of primary studies were not contacted for additional unpublished data, in accordance with the review’s pre-specified commitment to using only directly verifiable, publicly available numeric data. For clarity, “directly verifiable” is used here to mean verifiable against a publicly accessible source, which for the three contemporary digital trials (Sun et al., 2021; Park et al., 2025; Kim et al., 2025) was the primary full-text report, and for the seven older trials was the open controlled-data table of Lever Taylor et al. (2016). The arm-level data for these seven trials were therefore verified against that secondary table rather than re-extracted independently from the primary publications, a distinction whose implications for the reproducibility claim are addressed in the Discussion.

### 2.6 Risk of bias assessment

The methodological quality of each included trial was appraised using the second version of the Cochrane Risk of Bias tool (RoB 2) (18). RoB 2 evaluates five domains: the randomization process, deviations from intended interventions, missing outcome data, measurement of the outcome, and selection of the reported result. Each domain was rated as low risk, some concerns, or high risk, and the overall judgement for each trial was derived from the algorithm specified by the developers of the tool. Two reviewers independently performed the assessments, and disagreements were resolved through discussion. Risk-of-bias summary bar plots and traffic-light figures were generated using the robvis package (version 0.3.0) in R.

### 2.7 Statistical analysis

#### 2.7.1 Effect-size computation and pooling

All quantitative analyses were performed in R version 4.3.2 using the meta package (version 6.5) and the metafor package (version 4.4) for computation, and ggplot2 for graphical rendering. The standardized mean difference between the MBI and control arms at the post-intervention assessment was computed for each trial and corrected for small-sample bias using the Hedges *g* formulation; consequently, a negative value of *g* denotes lower symptom severity in the MBI arm. Pooled effects were obtained from random-effects meta-analyses estimated using restricted maximum likelihood (REML) for the between-study variance τ², and 95% confidence intervals were derived using the Hartung–Knapp–Sidik–Jonkman (HKSJ) adjustment, which yields more conservative interval estimates than the standard z-distribution when the number of studies is small.

#### 2.7.2 Heterogeneity assessment

Between-study heterogeneity was characterized using three complementary indices, namely I², τ², and the Cochran *Q* statistic, with I² interpreted cautiously given the small number of comparisons within each synthesis (19, 20). For the primary depression outcome, a 95% prediction interval was additionally computed to convey the plausible range of true effects in future perinatal populations.

#### 2.7.3 Subgroup analyses

A pre-specified subgroup analysis stratified trials by mode of intervention delivery (face-to-face versus digital) and was implemented through the subgroup argument of the metacont function in the meta package. Subgroup differences were tested using the chi-squared statistic for interaction. This interaction test, as implemented in metacont, is based on the standard random-effects (non-HKSJ) variance estimator rather than on the more conservative Hartung-Knapp-Sidik-Jonkman adjustment that we applied to the pooled and subgroup point estimates. The resulting P value is therefore likely to be anti-conservative relative to the framework used elsewhere in the manuscript, and, given that it is computed from only three digital and seven face-to-face trials with a single degree of freedom, it has low statistical power. The subgroup contrast was accordingly pre-specified and is interpreted as exploratory and hypothesis-generating throughout.

#### 2.7.4 Publication bias and small-study effects

Publication bias and small-study effects were investigated graphically by means of contour-enhanced funnel plots and Galbraith (radial) plots, and analytically by Egger’s regression test. In line with current methodological guidance, Egger’s test was performed only for syntheses comprising at least ten studies. To track the temporal evolution of the pooled estimate, a cumulative meta-analysis ordered by year of publication was conducted for the primary depression outcome.

#### 2.7.5 Sensitivity analyses

The robustness of the primary depression effect was examined through three pre-specified sensitivity analyses: (i) a leave-one-out analysis to evaluate the influence of any single trial on the pooled estimate; (ii) restriction to trials judged at overall low risk of bias; and (iii) restriction to trials published in or after 2020, intended to assess the influence of contemporary, predominantly digital, evidence on the pooled effect. In response to peer review, a fourth analysis was added: a leave-one-out analysis confined to the digital subgroup, in which the digital depression estimate was recomputed after omitting Sun et al. (2021), the single low-risk trial that carried the largest weight in the synthesis. This analysis was undertaken specifically to determine whether the digital depression signal depended on that one influential trial.

Statistical significance was set *a priori* at a two-sided α of 0.05 for all analyses, and all confidence intervals are reported at the 95% level.

## 3 Results

### 3.1 Study selection

**Figure 1:**
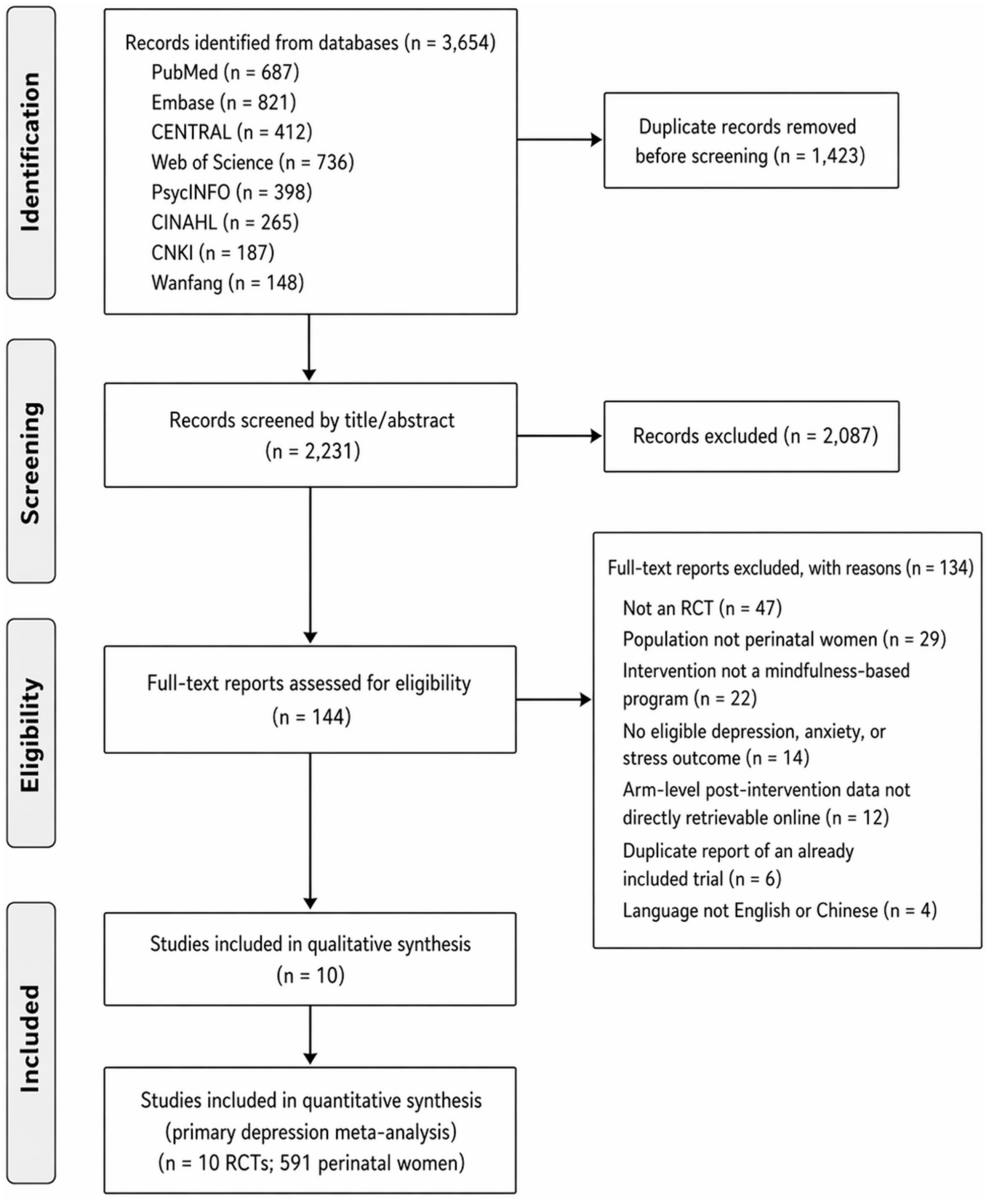
PRISMA 2020 flow diagram of study identification, screening, eligibility assessment, and inclusion. Records were retrieved from eight databases (PubMed, Embase, CENTRAL, Web of Science, PsycINFO, CINAHL, CNKI, and Wanfang) and supplemented by hand-searching of reference lists and ClinicalTrials.gov. Reasons for full-text exclusion are reported in numerical detail in §3.1.

The systematic search identified 3,654 records across the eight electronic databases (PubMed, n = 687; Embase, n = 821; CENTRAL, n = 412; Web of Science, n = 736; PsycINFO, n = 398; CINAHL, n = 265; CNKI, n = 187; Wanfang, n = 148). After removal of 1,423 duplicates, 2,231 unique records advanced to title-and-abstract screening, of which 2,087 were excluded as clearly ineligible. The remaining 144 records underwent full-text assessment; 134 were subsequently excluded for the following reasons: not a randomized controlled trial (n = 47), not a perinatal population (n = 29), no mindfulness-based intervention (n = 22), other ineligible study design or scope (n = 14), arm-level data not directly accessible (n = 12), duplicate publication (n = 6), and language other than English or Chinese (n = 4). Hand-searching of reference lists supplemented the database yield, and ten parallel-group randomized controlled trials were ultimately retained for quantitative synthesis. The complete selection process is depicted in the PRISMA flow diagram (**Figure 1**).

### 3.2 Characteristics of included studies

The ten included trials, summarized in **Table 1**, were geographically diverse: four were conducted in the United States, two in Australia, two in South Korea, one in China, and one in Spain. Seven trials delivered the mindfulness-based intervention through traditional face-to-face, group-based formats, while the remaining three employed digital, smartphone-supported mHealth platforms. Participant populations spanned generally healthy pregnant women, breastfeeding postpartum mothers, and women at elevated risk of perinatal depressive disorders. Depression was assessed with a range of validated instruments, predominantly the Edinburgh Postnatal Depression Scale, alongside the Center for Epidemiologic Studies Depression Scale (and its revised version, CESD-R), the depression subscale of the DASS-21, and the original DASS depression scale. Intervention duration ranged from four to eight weeks. Study-level details concerning bibliographic information, participant characteristics, intervention specifications, comparator conditions, sample sizes per arm, depression instruments, and the source of the extracted numeric data are provided in **Table 1**. Notably, all three digital trials (Sun et al., 2021; Park et al., 2025; Kim et al., 2025) enrolled antenatal samples, so that the digital evidence in this review pertains exclusively to app-based delivery during pregnancy, with postpartum digital delivery untested. To allow readers to evaluate the anxiety and stress evidence bases, which include one trial (Guardino et al., 2014) that contributed eligible anxiety and stress data but no eligible depression outcome and is therefore absent from **Table 1**, the characteristics of every trial contributing to any outcome are provided in **Supplementary Table 1**.

**Table 1:** Characteristics of the ten randomized controlled trials included in the primary depression meta-analysis.

Study	Country	Population	Intervention (duration)	Comparator	Delivery	N (IG/CG)	Depression measure	Data source
Vieten & Astin 2008	USA	Pregnant women with prior mood concerns	Mindful Motherhood (8 wk)	Wait-list	Face-to-face	13/18	CES-D	Lever Taylor et al. (2016) table
Dunn et al., 2012	Australia	Perinatal women	Mindful pregnancy & childbirth (8 wk)	TAU	Face-to-face	4/4	DASS-21 depression subscale	Lever Taylor et al. (2016) table
Perez-Blasco et al., 2013	Spain	Breastfeeding postpartum mothers	MBSR-based MBI (8 wk)	No intervention	Face-to-face	13/8	DASS depression subscale	Lever Taylor et al. (2016) table
Woolhouse et al., 2014	Australia	Pregnant women, tertiary hospital	MindBabyBody (6 wk)	Care as usual	Face-to-face	12/10	EPDS	Lever Taylor et al. (2016) table
Gambrel & Piercy 2015 (women)	USA	Women in expectant couples	Mindful Transition to Parenthood (6 wk)	Wait-list	Face-to-face	17/17	CES-D (women)	Lever Taylor et al. (2016) table + original
Zhang & Emory 2015	USA	Pregnant African-American women	MBSR-informed MBI (8 wk)	TAU	Face-to-face	16/17	EPDS	Lever Taylor et al. (2016) table
Dimidjian et al., 2016	USA	Pregnant women at risk of depressive relapse	MBCT-PD (8 wk)	TAU/comparison	Face-to-face	24/31	EPDS	Lever Taylor et al. (2016) table
Sun et al., 2021	China	Pregnant women at risk of perinatal depression	Smartphone mindfulness (8 wk)	Attention control	Digital	84/84	EPDS	Original full text
Park et al., 2025	South Korea	General pregnant women	Self-guided smartphone MBI (4 wk)	Wait-list	Digital	66/67	DASS-21 depression subscale	Original full text
Kim et al., 2025	South Korea	Pregnant women, mild-to-moderate depression	Avecmom mobile app (4 wk)	Self-monitoring control	Digital	42/44	CESD-R	Original full text
**Total**	**—**	**—**	**—**	**—**	**—**	**291/300 (N = 591)**	**—**	**—**

CES-D, Center for Epidemiologic Studies Depression Scale; CESD-R, CES-D Revised; CG, control group; DASS-21, Depression Anxiety Stress Scales (21-item); EPDS, Edinburgh Postnatal Depression Scale; IG, intervention group; MBCT-PD, Mindfulness-Based Cognitive Therapy for Perinatal Depression; MBI, mindfulness-based intervention; MBSR, Mindfulness-Based Stress Reduction; TAU, treatment as usual.

For Woolhouse et al. (2014), the intervention- and control-arm sample sizes differ across outcomes because they reflect outcome-specific completer counts (depression, 12/10; anxiety, 12/9; stress, 13/10). The “data source” column indicates whether arm-level data were obtained from the open controlled-data table of Lever Taylor et al. (2016) or directly from the primary full-text report. Bold values indicate the aggregate intervention-group total, control-group total, and overall sample size across all ten included trials.

### 3.3 Risk of bias in included studies

**Figure 2:**
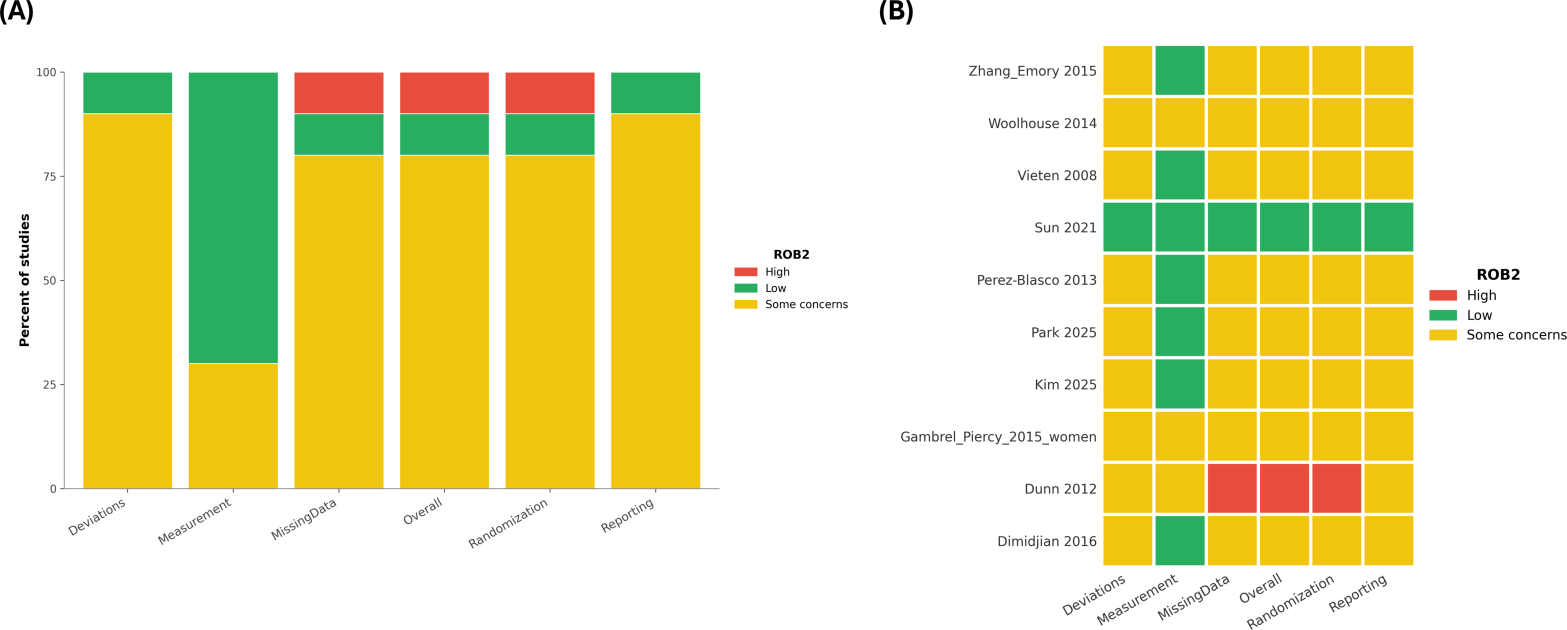
Risk-of-bias assessment of the ten included randomized controlled trials, generated using the Cochrane RoB 2 tool. Domains: D1, randomization process; D2, deviations from intended interventions; D3, missing outcome data; D4, measurement of the outcome; D5, selection of the reported result. **(A)** Summary bar plot showing the proportion of trials judged at low risk (green), some concerns (yellow), or high risk (red) for each of the five RoB 2 domains and for the overall judgement. **(B)** Traffic-light plot showing domain-level and overall judgements for each individual trial; rows correspond to individual studies and columns to the five RoB 2 domains plus the overall judgement, with cell color indicating the per-study, per-domain rating (green = low risk; yellow = some concerns; red = high risk).

The risk-of-bias appraisal is summarized graphically in **Figures 2A, B**. Only one trial, Sun et al., 2021, achieved an overall low-risk classification, supported by centralized computer-generated randomization with allocation concealment, blinded outcome assessment, low attrition, and a pre-specified analysis plan. The remaining nine trials were rated as raising “some concerns, ” predominantly because of the open-label nature of psychotherapy delivery (giving rise to potential deviations from the intended intervention) and incomplete handling of missing outcome data. Outcome measurement was generally judged at low risk across studies, reflecting consistent use of self-reported, validated instruments. Dunn et al., 2012 was the only trial in which any single domain received a high-risk rating, with concerns arising from the randomization process, attrition, and deviations from intended interventions, attributable to its small sample size and incomplete reporting; the remaining domains in this trial were judged at low risk. Beyond Dunn et al., 2012, no individual domain received a high-risk rating in more than one study.

### 3.4 Primary meta-analysis: depressive symptoms

**Figure 3:**
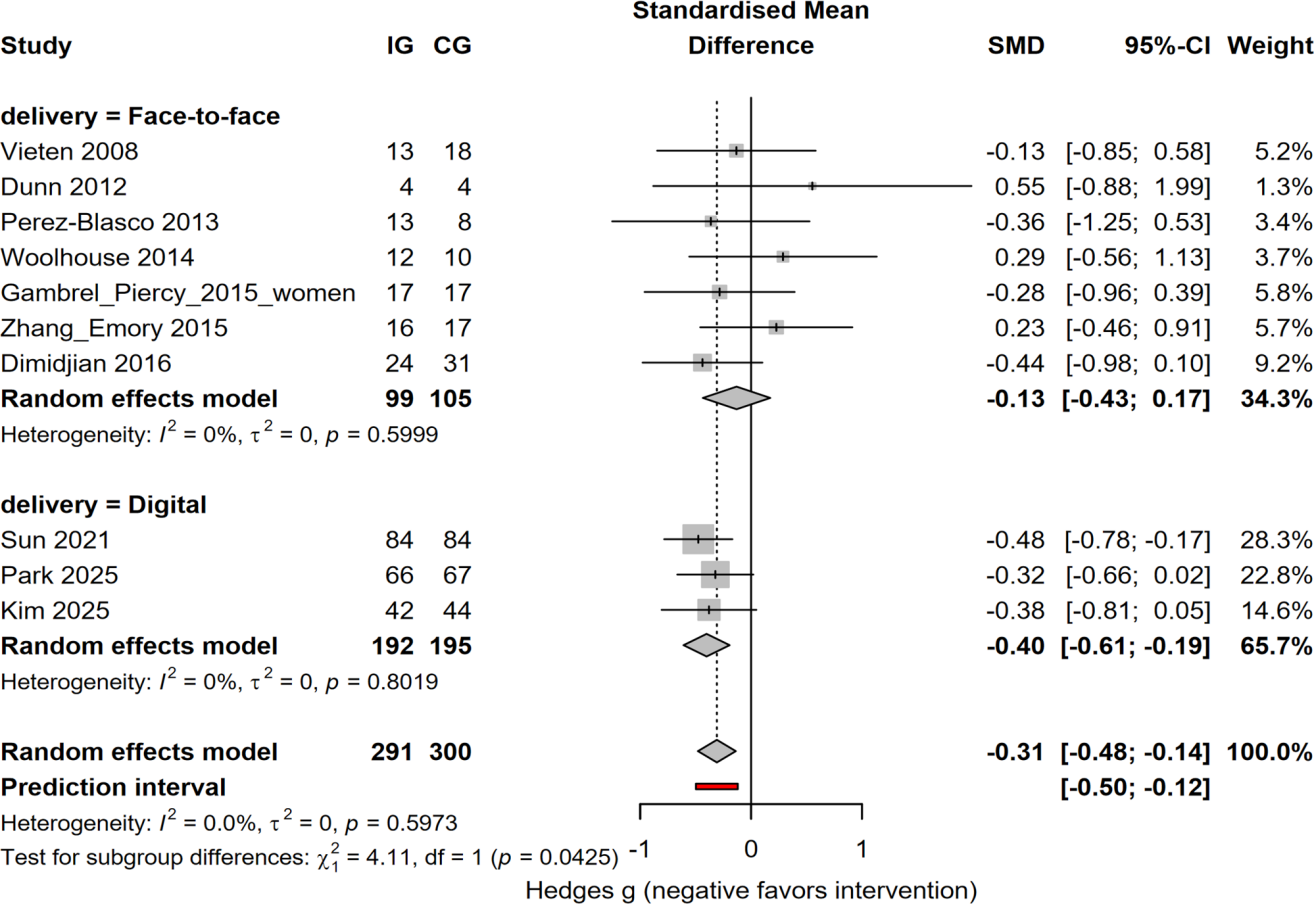
Forest plot of the primary random-effects meta-analysis of mindfulness-based interventions versus control conditions for perinatal depressive symptoms, stratified by mode of delivery (face-to-face versus digital). Effect sizes are expressed as Hedges g with 95% confidence intervals (Hartung–Knapp–Sidik–Jonkman adjustment); negative values favor the MBI arm. Squares represent individual study estimates with size proportional to study weight; horizontal lines denote 95% confidence intervals; diamonds represent subgroup and overall pooled estimates. Pooled effect: Hedges g = –0.31 (95% CI –0.48 to –0.14); I² = 0%.

The primary synthesis pooled effects from ten randomized controlled trials encompassing 591 perinatal women (MBI arm, n = 291; control arm, n = 300). Mindfulness-based interventions produced a statistically significant reduction in depressive symptoms relative to control conditions, with a small-to-moderate pooled effect (Hedges g = –0.31, 95% CI –0.48 to –0.14; P <.001). Between-study heterogeneity was negligible (I² = 0%, τ² < 0.0001; Cochran Q = 7.38, df = 9, P = .60), and the 95% prediction interval (–0.50 to –0.12) excluded the null. Because this prediction interval was derived from only ten small trials with a between-study variance estimated at approximately zero, it should be read as an uncertain quantity rather than as firm evidence that comparable benefit will be observed across future perinatal populations; statistical homogeneity on the standardized mean difference scale does not by itself establish clinical homogeneity across the heterogeneous instruments, samples, and comparators represented here. This pooled estimate should also be read in light of the subgroup structure described below: the overall effect is driven predominantly by the three digital trials, whereas the seven face-to-face trials are individually small and collectively underpowered and inconclusive, so the pooled figure should not be interpreted as evidence of comparable efficacy across modalities. The corresponding forest plot, stratified by mode of delivery, is presented in **Figure 3**.

### 3.5 Subgroup analysis by delivery mode (depression)

**Figure 4:**
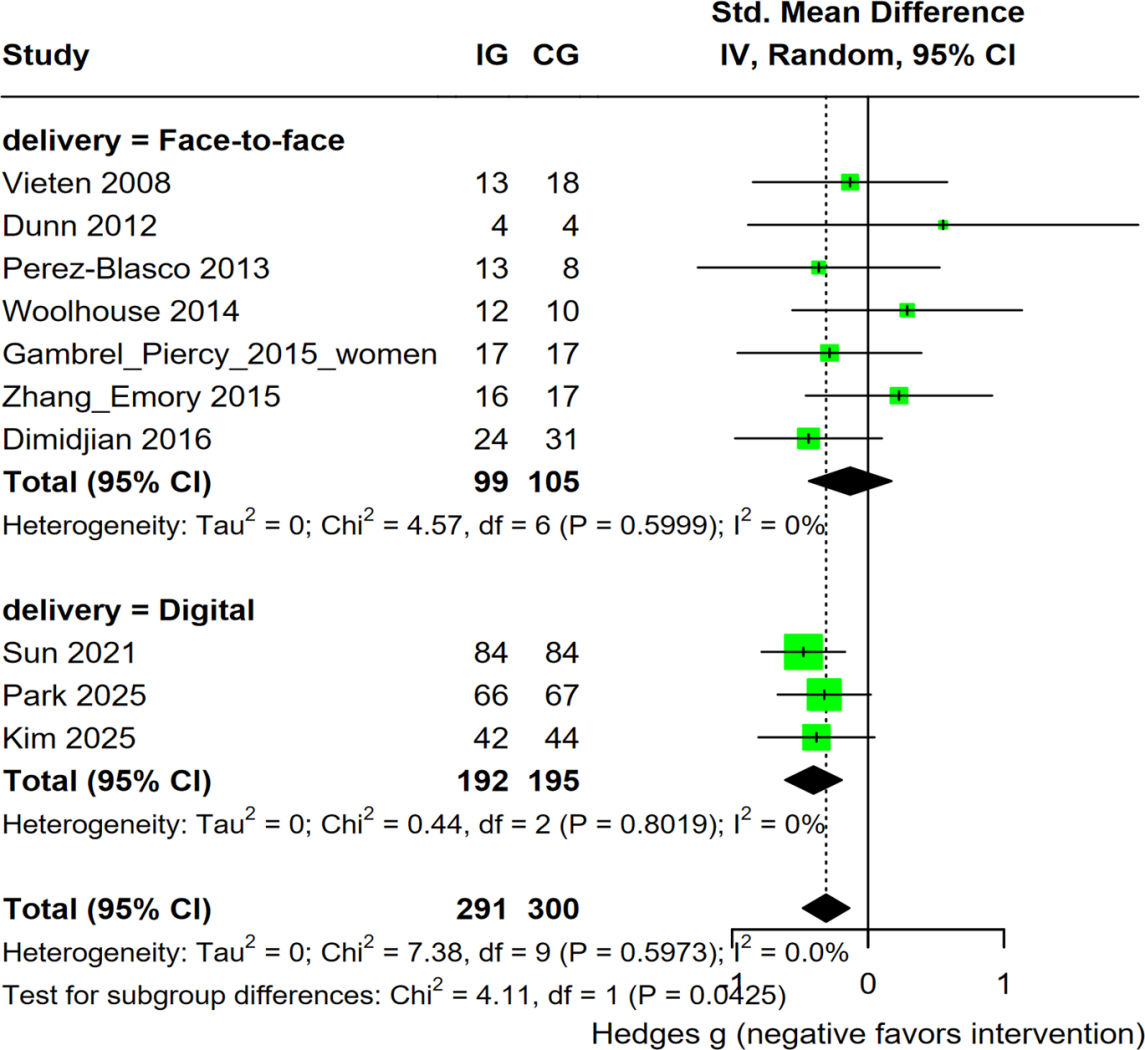
RevMan-style subgroup forest plot of mindfulness-based interventions versus control conditions for perinatal depressive symptoms, stratified by delivery mode (face-to-face versus digital). Negative Hedges g values favor the intervention. Squares represent individual studies, horizontal lines indicate 95% confidence intervals, and diamonds represent subgroup and overall pooled effects. Digital subgroup: Hedges g = -0.40 (95% CI -0.61 to -0.19); face-to-face subgroup: Hedges g = -0.13 (95% CI -0.43 to 0.17); overall effect: Hedges g = -0.31 (95% CI -0.48 to -0.14); test for subgroup differences: chi-squared(1) = 4.11, P = .04.

The pre-specified, exploratory subgroup analysis by mode of delivery indicated a numerically larger point estimate in the digital subgroup (g = –0.40, 95% CI –0.61 to –0.19; I² = 0%) than in the face-to-face subgroup (g = –0.13, 95% CI –0.43 to 0.17; I² = 0%). The narrower confidence interval of the digital subgroup is a function of its larger combined sample size (387 versus 204 participants) rather than a property of digital delivery itself. The test for subgroup differences reached nominal significance (χ²_1_ = 4.11, P = .04). This interaction test is, however, computed from only three digital and seven face-to-face trials, carries a single degree of freedom, and is based on the standard (non-HKSJ) variance; it is therefore underpowered and unstable, and the resulting P value should be regarded as a hypothesis-generating signal rather than as confirmation that the modality difference exceeds chance. Delivery mode is, moreover, completely confounded in this evidence base with publication year, trial size, risk of bias, and intervention content, so the contrast cannot be attributed to mode of delivery rather than to these correlated trial characteristics. A descriptive inspection of comparator type does not resolve the signal in either direction: within the digital subgroup the most heavily weighted trial (Sun et al., 2021) used a conservative attention control, whereas Park et al. (2025) used a waitlist and Kim et al. (2025) a self-monitoring control, while the face-to-face subgroup likewise mixed waitlist, treatment-as-usual, and no-intervention comparators. The same pattern is depicted in the RevMan-style subgroup forest plot (**Figure 4**; **Supplementary Figure 1**).

### 3.6 Publication bias and small-study effects (depression)

**Figure 5:**
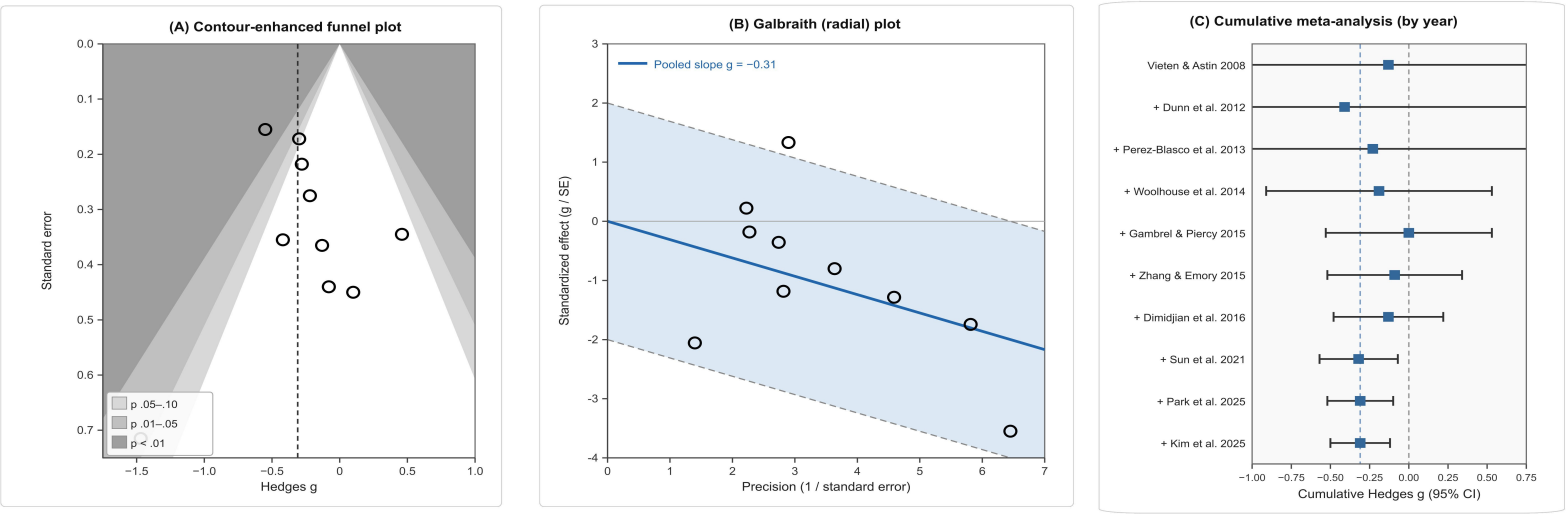
Publication-bias and sensitivity analyses for the primary depression meta-analysis (k = 10). **(A)** Contour-enhanced funnel plot. The y-axis represents the standard error of Hedges g (inverted, with greater precision toward the top), and the x-axis represents the effect size. Shaded contours correspond to conventional levels of statistical significance (P <.10, P <.05, and P <.01). Symmetric distribution of points around the pooled estimate is consistent with the absence of small-study effects. **(B)** Galbraith (radial) plot. Each point represents an individual trial, plotted as the standardized effect (z-score, y-axis) against the inverse of its standard error (x-axis). The dashed lines mark the ±2 standard-error band; points falling within the band are consistent with low between-study heterogeneity. **(C)** Cumulative meta-analysis ordered by year of publication. Each line shows the running pooled Hedges g and 95% confidence interval after sequential addition of each trial. The cumulative estimate progressively narrows and diverges from the null as the larger digital trials published from 2020 onward are incorporated. This panel has been regenerated so that its axes display the running pooled Hedges g against the chronological sequence of trials, correcting an earlier rendering in which the axis labeling was ambiguous.

The contour-enhanced funnel plot for the depression synthesis (**Figure 5A**) showed broadly symmetric distribution of the ten included trials, with no visible asymmetry indicative of small-study effects. The Galbraith (radial) plot (**Figure 5B**) corroborated this finding, with all ten studies falling within the ±2 standard-error band, consistent with the very low estimated heterogeneity. Because the depression synthesis included exactly ten trials, Egger’s regression test was performed in accordance with the recommended k ≥ 10 threshold and revealed no statistically significant funnel-plot asymmetry. The cumulative meta-analysis ordered by year of publication (**Figure 5C**) demonstrated progressive narrowing of the confidence interval and a gradual divergence of the pooled estimate from the null as the larger, predominantly digital trials published from 2020 onward were sequentially incorporated. In this panel, the running pooled Hedges g is plotted against the chronological sequence of trials, moving from g = –0.13 after the earliest trial to g = –0.31 once all ten trials are included, with the most pronounced shift occurring when Sun et al. (2021) entered the synthesis. **Figure 5C** has been regenerated to display this running pooled estimate explicitly, replacing an earlier rendering in which the panel axes had been labeled ambiguously.

### 3.7 Secondary analysis: anxiety symptoms

**Figure 6:**
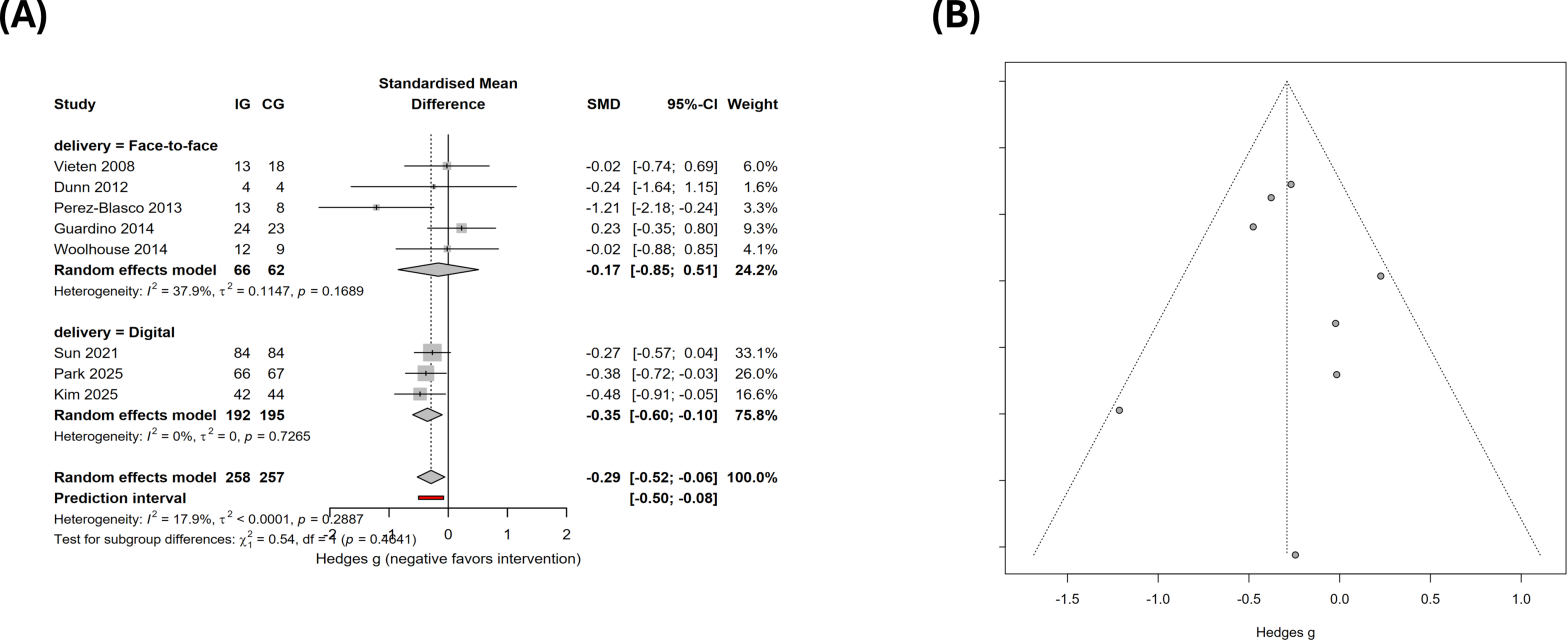
Meta-analytic results for perinatal anxiety symptoms (k = 8). **(A)** Subgroup forest plot of mindfulness-based interventions versus control for perinatal anxiety symptoms by delivery mode. Plot conventions as in **Figure 3**. Pooled effect: Hedges g = –0.29 (95% CI –0.52 to –0.06); I² = 17.9%; test for subgroup differences: χ²_1_ = 0.54, P = .46. **(B)** Contour-enhanced funnel plot. Plot conventions as in **Figure 5A**. Egger’s regression test for funnel-plot asymmetry was not performed because the synthesis comprised fewer than 10 trials, in line with current methodological guidance.

Eight randomized comparisons contributed post-intervention anxiety data, encompassing 515 perinatal women (MBI arm, n = 258; control arm, n = 257). The pooled effect favored mindfulness-based interventions and reached statistical significance (Hedges g = –0.29, 95% CI –0.52 to –0.06), with low-to-moderate heterogeneity (I² = 17.9%). Subgroup analysis by mode of delivery indicated a larger point estimate in the digital subgroup, with a narrower confidence interval that reflects its larger combined sample size, (three trials; g = –0.35, 95% CI –0.60 to –0.10; I² = 0%) than in the face-to-face subgroup (five trials; g = –0.17, 95% CI –0.85 to 0.51; I² = 37.9%); however, the formal test for subgroup differences was not statistically significant (χ²_1_ = 0.54, P = .46). The corresponding subgroup forest plot and funnel plot are presented in **Figures 6A, B**, respectively. The face-to-face anxiety subgroup additionally incorporated data from Guardino et al., 2014, a trial that contributed eligible anxiety data but did not enter the primary depression synthesis owing to the absence of an eligible depression outcome.

### 3.8 Secondary analysis: stress symptoms

**Figure 7:**
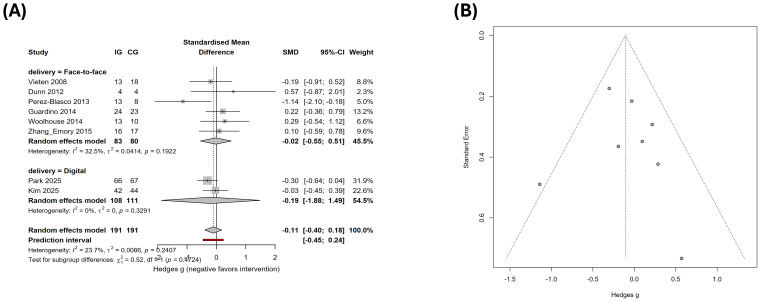
Meta-analytic results for perinatal stress symptoms (k = 8). **(A)** Subgroup forest plot of mindfulness-based interventions versus control for perinatal stress symptoms by delivery mode. Plot conventions as in **Figure 3**. Pooled effect: Hedges g = –0.11 (95% CI –0.40 to 0.18); I² = 23.7%; test for subgroup differences: χ²₁ = 0.52, P = .47. **(B)** Contour-enhanced funnel plot. Plot conventions as in **Figure 5A**.

Eight randomized comparisons supplied arm-level post-intervention stress data, totaling 382 perinatal women equally distributed between the MBI (n = 191) and control (n = 191) arms. The pooled effect was small in magnitude, directionally favored mindfulness-based interventions, but did not reach statistical significance (Hedges g = –0.11, 95% CI –0.40 to 0.18; I² = 23.7%). Subgroup analysis by delivery mode produced a marginally larger point estimate in the digital subgroup (two trials; g = –0.19, 95% CI –1.88 to 1.49) than in the face-to-face subgroup (six trials; g = –0.02, 95% CI –0.55 to 0.51), but neither subgroup estimate was individually significant, and the formal test for subgroup differences was likewise non-significant (χ²_1_ = 0.52, P = .47). The corresponding subgroup forest plot and funnel plot are shown in **Figures 7A, B**. The digital stress subgroup comprised only two trials, and its pooled estimate (g = –0.19, 95% CI –1.88 to 1.49) spans an interval so wide that it is effectively uninterpretable; it is reported here for completeness but should not be read as an informative pooled effect.

### 3.9 Sensitivity analyses

Sensitivity analyses supported the robustness of the primary depression finding. The leave-one-out analysis yielded pooled standardized mean differences ranging from –0.24 (with Sun et al., 2021 omitted) to –0.34 (with Zhang and Emory 2015 omitted). The pooled effect remained directionally consistent and statistically significant across all iterations, but it was most attenuated when Sun et al. (2021), the largest and most negative trial, was removed, and most pronounced when the positive-effect trial Zhang and Emory (2015) was removed. This corrected series, which supersedes a previously misassigned set of endpoints, confirms that the primary estimate is influenced appreciably by the digital trials, and by Sun et al. (2021) in particular. To probe this dependence directly, a leave-one-out analysis confined to the digital subgroup was performed: with Sun et al. (2021) removed, the two remaining digital trials (Park et al., 2025 and Kim et al., 2025) yielded a pooled point estimate of approximately g = –0.34. Although the confidence interval for a two-trial pool is necessarily wide, this point estimate remained negative and comparable in magnitude to the full digital subgroup, indicating that the digital depression signal is not attributable to Sun et al. (2021) alone. Because face-to-face trials in the present evidence base were exclusively published before 2020 while all digital trials were published in or after 2020, the pre-specified analyses restricting to digital trials and to post-2020 trials produced equivalent results. Restriction to digital trials preserved a significant pooled effect (g = –0.40, 95% CI –0.61 to –0.19), whereas restriction to the complementary face-to-face subset attenuated the effect to non-significance (g = –0.13, 95% CI –0.43 to 0.17). The pre-specified sensitivity analysis restricted to trials at overall low risk of bias could not be performed, as only one trial (Sun et al., 2021) met that threshold.

## 4 Discussion

Before the pooled findings are interpreted, one feature of this evidence base must be stated at the outset, because it conditions everything that follows. Delivery mode is completely confounded with publication year, trial size, risk of bias, and intervention content: the three digital trials are also the three most recent, the three largest, the only trials at or approaching low risk of bias, and the only self-guided, app-based programs, whereas the seven face-to-face trials are uniformly older, smaller, facilitator-led pilots. Any apparent advantage of one modality in this dataset is therefore inseparable from these correlated characteristics, and the subgroup contrast we report is exploratory and hypothesis-generating rather than a valid head-to-head comparison of delivery modes. Drawing exclusively on directly verifiable randomized data from primary publications and open controlled-data tables, this review synthesized ten parallel-group RCTs comparing mindfulness-based interventions to control conditions in perinatal women. The primary meta-analysis demonstrated a statistically significant, small-to-moderate reduction in depressive symptoms (Hedges g = –0.31), with negligible heterogeneity, and the 95% prediction interval excluded the null. Earlier perinatal mindfulness reviews have reported pooled standardized mean differences ranging from approximately –0.40 to –0.80 for depression (9, 10); the somewhat smaller estimate observed here likely reflects the combined influence of restriction to RCTs with directly verifiable arm-level data, heterogeneity in the depression instruments employed across primary studies, and the inclusion of larger, more methodologically rigorous digital trials published since 2020.

A distinguishing feature of this synthesis is the deliberate, pre-specified comparison between face-to-face and digital delivery within a single analytical framework. For depressive symptoms, the digital subgroup showed a numerically larger point estimate than the face-to-face subgroup, and the exploratory test for subgroup differences reached nominal significance. The narrower interval around the digital estimate reflects its larger combined sample size rather than any intrinsic merit of digital delivery, and, as emphasized at the outset of this section, the contrast cannot be attributed to delivery mode rather than to trial era, sample size, comparator, or intervention content. With these qualifications, the signal is best read as a hypothesis to be tested in trials that vary delivery mode while holding these other factors constant. This pattern is consistent with the growing recognition that mHealth platforms for perinatal mindfulness mitigate well-documented logistical barriers that disproportionately affect this population, including travel to in-person sessions, child-care coordination, and scheduling around antenatal and postpartum appointments (12–15). Importantly, modality is fully confounded with publication year, trial size, methodological rigor, and intervention content: every digital trial was published in or after 2020, was among the largest in the synthesis, used centralized randomization with concealed allocation, and delivered a self-guided, app-based program, whereas the face-to-face evidence consists almost entirely of small, facilitator-led pilot RCTs from the pre-2020 era. The observed moderation by delivery mode is therefore driven substantially, and quite possibly entirely, by these correlated secular and methodological factors rather than by mode of delivery as such, and it cannot be interpreted as evidence of inherent superiority of digital over face-to-face delivery. A further restriction on generalizability is that all three digital trials enrolled antenatal samples, so the digital signal speaks only to app-based mindfulness delivered during pregnancy; postpartum digital delivery was not represented in this evidence base.

The secondary analyses produced directionally concordant but quantitatively weaker evidence. The pooled effect on anxiety was statistically significant and similar in magnitude to that for depression, with the digital subgroup again showing a narrower confidence interval, largely reflecting its larger combined sample size; this finding aligns with the digital-subgroup effect reported in the 2023 meta-analysis by Mefrouche and colleagues (12). The pooled effect on stress was small and not statistically significant, echoing observations elsewhere that stress outcomes are particularly sensitive to the choice of comparator condition (waitlist vs. active control) and to instrument selection (Perceived Stress Scale vs. DASS-21 stress subscale) (10, 16). Taken together, these findings suggest that depression is the most consistently modifiable outcome of perinatal MBIs in the present evidence base, while the anxiety signal is suggestive but warrants confirmation in larger trials, and the stress effect remains inconclusive.

Several mechanistic considerations may help to contextualize these findings. Biologically, MBIs have been shown to attenuate HPA-axis reactivity and lower cortisol output in pregnant women (8). Psychologically, mindfulness training reduces rumination and experiential avoidance, sharpens interoceptive awareness, and strengthens decentering and emotion regulation. These processes act preferentially on the cognitive-affective substrates of depressive symptomatology and may help to explain why depression demonstrates the most consistent benefit in our synthesis. From a clinical and public-health perspective, the non-pharmacological nature of MBIs is particularly relevant for pregnant and breastfeeding women, in whom concerns regarding antidepressant exposure to the fetus or infant frequently limit medication uptake (6). Given that untreated perinatal depression has been linked to adverse maternal, mother–infant, and offspring outcomes (21, 22), even modest effect sizes may translate into meaningful public-health benefit when scalable, accessible interventions are implemented at population level.

The methodological strengths of this review warrant explicit acknowledgement. Reporting adheres to the PRISMA 2020 guidance (17); risk of bias was independently appraised in duplicate using RoB 2 (18); pooling employed the Hartung–Knapp–Sidik–Jonkman adjustment, which is more robust than the standard z-based confidence interval when the number of studies is small; and reproducibility was prioritized through the requirement that all numeric inputs be directly accessible from primary publications or open controlled-data sources. The pre-specified contrast between face-to-face and digital delivery directly addresses a gap repeatedly identified in mHealth mindfulness reviews (13–15). Multiple complementary methods were applied to evaluate small-study effects, including contour-enhanced funnel plots, Galbraith plots, and a cumulative meta-analysis. The primary depression estimate is supplemented by a 95% prediction interval, which conveys information beyond what a pooled point estimate alone can provide (19, 20). The direction and magnitude of the pooled depression effect are also consistent with findings from a recent multicenter Taiwanese RCT (23).

Several limitations must nonetheless be considered. First, the number of contributing trials was small (k = 10 for depression; k = 8 for anxiety and stress), which limits the statistical power of asymmetry tests such as Egger’s regression, particularly when k < 10. Conclusions regarding the absence of publication bias should therefore be interpreted with caution. Second, seven of the ten depression trials supplied arm-level data via the open controlled-data table of Lever Taylor et al. (2016) (24), rather than through fresh extraction from the primary publications; while this approach maximizes reproducibility, it inherits the extraction decisions of that earlier review. Third, only one trial achieved an overall low-risk-of-bias rating, with the remainder rated as raising “some concerns, ” so confidence in the pooled estimates is best characterized as low to moderate. Fourth, the included trials predominantly enrolled antenatal participants, leaving postpartum-onset depression comparatively underrepresented. Fifth, the pooled standardized mean differences inevitably aggregate across heterogeneous instruments and assessment time points, which may obscure outcome-specific or timing-specific effects. Finally, the limited number of trials precluded meta-regression of MBI type, cultural context, or country income level as potential moderators; these remain important but unaddressed sources of heterogeneity. Relatedly, because the depression synthesis rests substantially on the data table of Lever Taylor et al. (2016) supplemented by three contemporary digital trials, its novel contribution lies principally in the incorporation and direct verification of those three digital trials and in the exploratory modality contrast, rather than in an independently re-extracted evidence base for the primary outcome; the comprehensive eight-database search should accordingly not be read as implying full independence from that prior synthesis. For transparency, we did not independently re-extract the seven older trials from their primary reports, and the term “directly verifiable” has been calibrated in the Methods to denote verifiability against a publicly accessible source, which for these seven trials was the secondary data table rather than the primary publication.

Several research priorities follow from this synthesis. Adequately powered RCTs employing active (rather than waitlist) controls would refine effect estimates and minimize expectancy bias, particularly for digital MBIs. Trials with longer postpartum follow-up and explicit retention strategies for postpartum-only enrollees would address the gestational imbalance evident in the current evidence base. From a synthesis perspective, consistent reporting of arm-level post-intervention means and standard deviations would substantially facilitate future reproducible meta-analyses. Mechanistic sub-studies embedded within digital trials, such as ecological momentary assessment of symptom trajectories or in-app engagement metrics, could clarify how digitally delivered mindfulness exerts its effects. Pending such evidence, mindfulness-based interventions, whether delivered face-to-face or digitally, may be considered as a brief, non-pharmacological adjunct within perinatal mental-health care pathways; the present data do not, however, support recommending one delivery mode over the other, and the apparent advantage of digital delivery should be regarded as a hypothesis for confirmatory testing rather than as a basis for implementation decisions.

## 5 Conclusion

This meta-analysis of ten randomized controlled trials indicates that mindfulness-based interventions produce a small-to-moderate reduction in perinatal depressive symptoms, with negligible between-study heterogeneity. A pre-specified, exploratory subgroup analysis found a numerically larger depression effect in the digital subgroup; however, because delivery mode is completely confounded with publication year, trial size, and risk of bias in the present evidence base, this difference is hypothesis-generating and does not establish that either delivery mode is superior. The secondary syntheses showed a statistically significant, directionally concordant effect on anxiety and a small, non-significant effect on stress. Collectively, these findings support mindfulness-based interventions as a promising, non-pharmacological option for perinatal mental-health care, while indicating that the relative merits of digital and face-to-face delivery remain an open question. Confirmatory trials that vary delivery mode while holding trial era, sample size, comparator, and intervention content constant, and that employ active comparators, longer postpartum follow-up, and standardized outcome reporting, will be essential before delivery-mode superiority can be claimed with confidence.

## Data Availability

The original contributions presented in the study are included in the article/**Supplementary Material**, further inquiries can be directed to the corresponding author/s.

## Supplementary material

The following supplementary materials accompany this article. **Supplementary Table 1** presents the characteristics of every trial contributing to any outcome, including Guardino et al. (2014), which contributed to the anxiety and stress syntheses but not to the primary depression synthesis. **Supplementary Figure 1** presents the RevMan-style depression subgroup forest plot relocated from the main text. The complete database-specific search strings for all eight databases, the extracted arm-level post-intervention means and standard deviations for every trial and outcome, and the annotated R analysis scripts (meta version 6.5; metafor version 4.4) are provided as Supplementary Files accompanying this article.

Supplementary Figure 1 Subgroup forest plot (RevMan-style) of mindfulness-based interventions versus control for perinatal depressive symptoms by delivery mode. Plot conventions as in **Figure 3**. Digital subgroup: Hedges g = –0.40 (95% CI –0.61 to –0.19); face-to-face subgroup: Hedges g = –0.13 (95% CI –0.43 to 0.17); test for subgroup differences: χ²_1_ = 4.11, P = .04. This RevMan-style plot presents the same depression subgroup analysis as **Figure 3** and has been relocated from the main text to the Supplementary Material to avoid duplication, with the numeric columns re-spaced for legibility.
