# Supplementary material for: Mindfulness-based interventions for perinatal depression, anxiety, and stress: a systematic review and meta-analysis of randomized controlled trials, with an exploratory subgroup signal by delivery mode
Source: Front Psychiatry. 2026 Aug 4;17:1868153. doi: 10.3389/fpsyt.2026.1868153 (PMC13481379; doi:10.3389/fpsyt.2026.1868153)
Supplement: Supplementary file 1 [file SupplementaryFile2.docx]

**Supplementary File S2. Database-specific search strategies**

This file reports the complete database-specific search strategies for the eight bibliographic databases searched, together with the ClinicalTrials.gov supplementary search and the hand-search procedure. Each strategy combines a population block, an intervention block (core mindfulness terms plus a digital/mHealth supplement), and an outcome block with the Boolean operator AND, with a validated randomized controlled trial filter applied where the database offered one. Search date: 28 February 2026. Date limits: 1 January 2008 to 28 February 2026. Language limits: English or Chinese. Eligible design: parallel-group randomized controlled trials. Total records retrieved across the eight databases: 3,654.

**1. PubMed**

Reported record count: 687

((perinatal[tiab] OR pregnancy[tiab] OR pregnant[tiab] OR antenatal[tiab] OR prenatal[tiab] OR postpartum[tiab] OR postnatal[tiab] OR maternal[tiab])

AND

(mindfulness[tiab] OR mindful[tiab] OR MBSR[tiab] OR MBCT[tiab] OR MBCP[tiab] OR meditation[tiab] OR app[tiab] OR mobile[tiab] OR digital[tiab] OR mHealth[tiab] OR "web-based"[tiab] OR online[tiab] OR eHealth[tiab] OR internet[tiab] OR smartphone[tiab])

AND

(depression[tiab] OR depressive[tiab] OR anxiety[tiab] OR anxious[tiab] OR stress[tiab] OR distress[tiab])

AND

(randomized[tiab] OR randomised[tiab] OR randomly[tiab] OR trial[tiab] OR RCT[tiab] OR "randomized controlled trial"[Publication Type] OR "controlled clinical trial"[Publication Type]))

AND ("2008/01/01"[Date - Publication] : "2026/02/28"[Date - Publication])

AND (english[Language] OR chinese[Language])

**2. Embase**

Reported record count: 821

('perinatal':ti,ab OR 'pregnancy':ti,ab OR 'pregnant':ti,ab OR 'antenatal':ti,ab OR 'prenatal':ti,ab OR 'postpartum':ti,ab OR 'postnatal':ti,ab OR 'maternal':ti,ab)

AND

('mindfulness':ti,ab OR 'mindful':ti,ab OR 'mbsr':ti,ab OR 'mbct':ti,ab OR 'mbcp':ti,ab OR 'meditation':ti,ab OR 'app':ti,ab OR 'mobile':ti,ab OR 'digital':ti,ab OR 'mhealth':ti,ab OR 'web-based':ti,ab OR 'online':ti,ab OR 'ehealth':ti,ab OR 'internet':ti,ab OR 'smartphone':ti,ab)

AND

('depression':ti,ab OR 'depressive':ti,ab OR 'anxiety':ti,ab OR 'stress':ti,ab OR 'distress':ti,ab)

AND

('randomized controlled trial'/exp OR 'controlled clinical trial'/exp OR random*:ti,ab OR randomised:ti,ab OR randomized:ti,ab OR rct:ti,ab OR trial:ti,ab)

AND [2008-2026]/py

AND ([english]/lim OR [chinese]/lim)

**3. Cochrane CENTRAL**

Reported record count: 412

(perinatal OR pregnancy OR pregnant OR antenatal OR prenatal OR postpartum OR postnatal OR maternal):ti,ab,kw

AND

(mindfulness OR mindful OR MBSR OR MBCT OR MBCP OR meditation OR app OR mobile OR digital OR mHealth OR web-based OR online OR eHealth OR internet OR smartphone):ti,ab,kw

AND

(depression OR depressive OR anxiety OR stress OR distress):ti,ab,kw

Publication year from 2008 to 2026

**4. Web of Science Core Collection**

Reported record count: 736

TS=((perinatal OR pregnancy OR pregnant OR antenatal OR prenatal OR postpartum OR postnatal OR maternal)

AND

(mindfulness OR mindful OR MBSR OR MBCT OR MBCP OR meditation OR app OR mobile OR digital OR mHealth OR "web-based" OR online OR eHealth OR internet OR smartphone)

AND

(depression OR depressive OR anxiety OR stress OR distress)

AND

(randomized OR randomised OR randomly OR RCT OR trial OR "controlled trial"))

Timespan: 2008-2026

Languages: English OR Chinese

Document types: Article

**5. PsycINFO**

Reported record count: 398

((TI,AB(perinatal OR pregnancy OR pregnant OR antenatal OR prenatal OR postpartum OR postnatal OR maternal))

AND

(TI,AB(mindfulness OR mindful OR MBSR OR MBCT OR MBCP OR meditation OR app OR mobile OR digital OR mHealth OR "web-based" OR online OR eHealth OR internet OR smartphone))

AND

(TI,AB(depression OR depressive OR anxiety OR stress OR distress))

AND

(TI,AB(randomized OR randomised OR randomly OR RCT OR trial OR "controlled trial")))

Limits: 2008-2026; English or Chinese; peer-reviewed journal articles

**6. CINAHL**

Reported record count: 265

((TI perinatal OR AB perinatal OR TI pregnancy OR AB pregnancy OR TI pregnant OR AB pregnant OR TI antenatal OR AB antenatal OR TI prenatal OR AB prenatal OR TI postpartum OR AB postpartum OR TI postnatal OR AB postnatal OR TI maternal OR AB maternal)

AND

(TI mindfulness OR AB mindfulness OR TI mindful OR AB mindful OR TI MBSR OR AB MBSR OR TI MBCT OR AB MBCT OR TI MBCP OR AB MBCP OR TI meditation OR AB meditation OR TI app OR AB app OR TI mobile OR AB mobile OR TI digital OR AB digital OR TI mHealth OR AB mHealth OR TI web-based OR AB web-based OR TI online OR AB online OR TI eHealth OR AB eHealth OR TI internet OR AB internet OR TI smartphone OR AB smartphone)

AND

(TI depression OR AB depression OR TI depressive OR AB depressive OR TI anxiety OR AB anxiety OR TI stress OR AB stress OR TI distress OR AB distress)

AND

(TI randomized OR AB randomized OR TI randomised OR AB randomised OR TI randomly OR AB randomly OR TI RCT OR AB RCT OR TI trial OR AB trial OR TI controlled trial OR AB controlled trial))

Limits: 2008-2026; English or Chinese; peer-reviewed

**7. CNKI**

Reported record count: 187

主题 = (围产期 OR 妊娠 OR 孕期 OR 孕妇 OR 产前 OR 产后 OR 母体 OR perinatal OR pregnancy OR pregnant OR antenatal OR prenatal OR postpartum OR postnatal OR maternal)

AND

主题 = (正念 OR 正念减压 OR 正念认知疗法 OR 冥想 OR MBSR OR MBCT OR MBCP OR mindfulness OR mindful OR meditation OR 手机 OR 应用程序 OR app OR 移动 OR 数字 OR mHealth OR 网络 OR 在线 OR 互联网 OR smartphone)

AND

主题 = (抑郁 OR 焦虑 OR 压力 OR 应激 OR depression OR anxiety OR stress)

AND

主题 = (随机 OR 随机对照 OR 临床试验 OR randomized OR randomised OR trial OR RCT)

Publication years: 2008-2026

Languages: Chinese or English

**8. Wanfang Data**

Reported record count: 148

主题: (围产期 OR 妊娠 OR 孕期 OR 孕妇 OR 产前 OR 产后 OR 母体 OR perinatal OR pregnancy OR pregnant OR antenatal OR prenatal OR postpartum OR postnatal OR maternal)

AND

主题: (正念 OR 正念减压 OR 正念认知疗法 OR 冥想 OR MBSR OR MBCT OR MBCP OR mindfulness OR mindful OR meditation OR 手机 OR 应用程序 OR app OR 移动 OR 数字 OR mHealth OR 网络 OR 在线 OR 互联网 OR smartphone)

AND

主题: (抑郁 OR 焦虑 OR 压力 OR 应激 OR depression OR anxiety OR stress)

AND

主题: (随机 OR 随机对照 OR 临床试验 OR randomized OR randomised OR trial OR RCT)

Publication years: 2008-2026

Languages: Chinese or English

**9. ClinicalTrials.gov supplementary search**

Search terms:

(perinatal OR pregnancy OR pregnant OR antenatal OR prenatal OR postpartum OR postnatal OR maternal)

AND

(mindfulness OR mindful OR MBSR OR MBCT OR MBCP OR meditation OR app OR mobile OR digital OR mHealth OR web-based OR online OR eHealth OR internet OR smartphone)

Condition/disease:

(depression OR anxiety OR stress)

Study type:

Interventional

Status:

Completed

Date range:

First posted or results available from 1 January 2008 to 28 February 2026

**10. Hand-searching**

The reference lists of every included primary study and relevant systematic reviews/meta-analyses were hand-searched.

Potentially eligible records were screened against the same PICOS criteria as database records.
