# Supplementary material for: Mindfulness-based interventions for perinatal depression, anxiety, and stress: a systematic review and meta-analysis of randomized controlled trials, with an exploratory subgroup signal by delivery mode
Source: Front Psychiatry. 2026 Aug 4;17:1868153. doi: 10.3389/fpsyt.2026.1868153 (PMC13481379; doi:10.3389/fpsyt.2026.1868153)
Supplement: Supplementary file 2 [file Table1.docx]

**Supplementary Material**

**Supplementary Table S1.** Characteristics of every randomized controlled trial contributing to any outcome (depression, anxiety, or stress). Guardino et al. (2014) contributed eligible anxiety and stress data but no eligible depression outcome and therefore does not appear in main-text Table 1.

| **Study** | **Country** | **Population** | **Intervention (duration)** | **Comparator** | **Delivery** | **Outcomes contributed: n (IG/CG)** | **Instruments (D / A / S)** | **Data source** |
| --- | --- | --- | --- | --- | --- | --- | --- | --- |
| Vieten & Astin 2008 | USA | Pregnant women with prior mood concerns | Mindful Motherhood (8 wk) | Wait-list | Face-to-face | D 13/18; A 13/18; S 13/18 | CES-D / STAI / PSS | Lever Taylor et al. (2016) table |
| Dunn et al. 2012 | Australia | Perinatal women | Mindful pregnancy & childbirth (8 wk) | TAU | Face-to-face | D 4/4; A 4/4; S 4/4 | DASS-21 (D/A/S) | Lever Taylor et al. (2016) table |
| Perez-Blasco et al. 2013 | Spain | Breastfeeding postpartum mothers | MBSR-based MBI (8 wk) | No intervention | Face-to-face | D 13/8; A 13/8; S 13/8 | DASS (orig.) (D/A/S) | Lever Taylor et al. (2016) table |
| Guardino et al. 2014 | USA | Pregnant women with elevated stress | Mindful Awareness Practices (6 wk) | Reading/active control | Face-to-face | A 24/23; S 24/23 | STAI / PSS | Lever Taylor et al. (2016) table |
| Woolhouse et al. 2014 | Australia | Pregnant women, tertiary hospital | MindBabyBody (6 wk) | Care as usual | Face-to-face | D 12/10; A 12/9; S 13/10 | EPDS / STAI / PSS | Lever Taylor et al. (2016) table |
| Gambrel & Piercy 2015 (women) | USA | Women in expectant couples | Mindful Transition to Parenthood (6 wk) | Wait-list | Face-to-face | D 17/17 | CES-D (women) | Lever Taylor et al. (2016) table + original |
| Zhang & Emory 2015 | USA | Pregnant African-American women | MBSR-informed MBI (8 wk) | TAU | Face-to-face | D 16/17; S 16/17 | EPDS / PSS | Lever Taylor et al. (2016) table |
| Dimidjian et al. 2016 | USA | Pregnant women at risk of depressive relapse | MBCT-PD (8 wk) | TAU / comparison | Face-to-face | D 24/31 | EPDS | Lever Taylor et al. (2016) table |
| Sun et al. 2021 | China | Pregnant women at risk of perinatal depression | Smartphone mindfulness (8 wk) | Attention control | Digital | D 84/84; A 84/84 | EPDS / GAD-7 | Original full text |
| Park et al. 2025 | South Korea | General pregnant women | Self-guided smartphone MBI (4 wk) | Wait-list | Digital | D 66/67; A 66/67; S 66/67 | DASS-21 (D/A/S) | Original full text |
| Kim et al. 2025 | South Korea | Pregnant women, mild-to-moderate depression | Avecmom mobile app (4 wk) | Self-monitoring control | Digital | D 42/44; A 42/44; S 42/44 | CESD-R / GAD-7 / PSS | Original full text |

*Abbreviations: A, anxiety; CES-D, Center for Epidemiologic Studies Depression Scale; CESD-R, CES-D Revised; CG, control group; D, depression; DASS, Depression Anxiety Stress Scales; DASS-21, 21-item DASS; EPDS, Edinburgh Postnatal Depression Scale; GAD-7, Generalized Anxiety Disorder 7-item scale; IG, intervention group; MBCT-PD, Mindfulness-Based Cognitive Therapy for Perinatal Depression; MBI, mindfulness-based intervention; MBSR, Mindfulness-Based Stress Reduction; PSS, Perceived Stress Scale; S, stress; STAI, State-Trait Anxiety Inventory; TAU, treatment as usual.*

*Note.* Arm sizes are given per outcome and reflect outcome-specific completer counts; where these differ within a trial (for example, Woolhouse et al. 2014), the difference is attributable to outcome-specific completion rather than to differing randomization.

**Supplementary Figure S1.** RevMan-style subgroup forest plot of mindfulness-based interventions versus control for perinatal depressive symptoms by delivery mode (the same depression subgroup analysis shown in main-text Figure 3), relocated from the main text and regenerated with the numeric columns re-spaced for legibility. The figure file accompanies this submission.

**Additional deposited materials.** The complete database-specific search strategies for all eight databases (Supplementary File S2), the extracted arm-level post-intervention means and standard deviations for every trial and outcome (Supplementary File S3), and the annotated R analysis scripts (Supplementary File S4; meta version 6.5; metafor version 4.4) are provided as Supplementary Files accompanying this article.
